# A heijunka study for the production of standard parts included in a customized finished product

**DOI:** 10.1371/journal.pone.0260515

**Published:** 2021-12-02

**Authors:** Paulina Rewers, Jacek Diakun

**Affiliations:** Department of Mechanical Engineering, Poznań University of Technology, Poznań, Poland; Univerza v Mariboru, SLOVENIA

## Abstract

Efficient order execution plays a crucial role in the activity of every company. In production planning it is important to find a balance between the fluctuations of orders and stability of production flow regarding the company. One of the methods of achieving this goal is heijunka (production leveling). This paper presents a study of choosing the best variant of the production planning and control system for the production of standard parts. Three variants are investigated regarding delays in order delivery. The analysis of variants was conducted using a simulation method. The method of choosing the best variant for the production system being investigated is also proposed. The results show that the best variant is a mix of production leveling and production "for stock".

## Introduction

In the era of constantly growing competition and a clear increase in customer requirements, production companies are trying to adapt to the changing economic conditions as muchas possible. Some place emphasis on continuous improvement of production processes in order to reduce production costs and increase the quality of the offered goods, for example by using Lean Manufacturing [1]. Others decide to manufacture individualized (customized) products that meet today’s customer requirements [2]. In product customization, it is important to deliver products tailored to individual requirements or customer concepts. Unfortunately, this strategy entails a number of problems related to the increase in the costs of product design and production [3]. Due to the increase in costs, many companies decide to market products in accordance with the concept of an assemble-to-order system, which provides customizable final products composed of standard parts that can be produced in a serial way. One of the many threats caused by this type of production are the constant changes in production plans for the components included in the finished product, dictated by changing orders for final products, or uneven inventory levels, as well as the load on machines [4, 5].

One of the ways of limiting the constant changes in the production plan and smoothing the flow of products in the supply chain is production leveling, which is considered a modern way of planning and controlling production. Nevertheless, the production leveling literature is not extensive. One can find only a few valuable items that present ways to implement production leveling, and a dozen or so that present research on the impact of production leveling on the production process. Production leveling (heijunka) is commonly regarded as a method of product sequencing in order to balance production, and increase productivity and flexibility by minimizing differences in workloads [6, 7]. According to the authors, production leveling consists of determining the order and size of the batch of manufactured products so that the current demand is met from the warehouse and does not cause sudden changes in the production plan [8–10]. However, production leveling is not applicable to every enterprise and process. The idea of production leveling is based on use of a repeatable and unchangeable production plan, in which products are produced in the smallest batches possible. This means frequent changeovers, but increases the availability of various finished products in stock. In the case of mass production, where production is most often carried out in a continuous manner, and the given workplaces continuously produce the same product, the use of leveled production is pointless. Also, in the case of unit production, in which all components included in the product are unique, leveled production is not possible. It is only possible to implement in the production of standard products, produced in a serial manner, for which the profitable and feasible stations have become a fixed and unchanging production plan [10].

In assembly to order, the production of parts included in the finished product, which are usually standard products, produced in a series, plays a particularly important role. This situation forces production companies to look for optimal solutions in the field of production planning and control. For this purpose, many of them, before introducing a specific solution, decide to apply simulation methods that allow for the analysis and evaluation of various variants of the introduced solutions. Simulation allows insight into complex process structures, tests new rules of production organization or the way materials flow through the process, analyzes production indicators or collects information and knowledge without disturbing the actual process [11, 12].

In the literature, examples of simulation methods used for leveled production can be found. Matzka et al. [13] used a queuing network model of a heijunka-controlled Kanban manufacturing system in order to find optimal buffer capacities. Kanban is a part of the Toyota Production System, which was created to control inventory levels, the production and supply of components, and in some cases, raw material. Checking the condition of materials in a production hall or warehouse is done with the help of a special kind of cards, for example production order, schedules, bill of materials, or product structure [14]. The demand was controlled and limited by a Kanban loop. Runkler [15] conducted a simulation comparative study of Kanban and heijunka controlled production systems of an electronic circuit manufacturer. The study showed the best strategy is a mix of Kanban and heijunka: Kanban is better for the startup phase of the process, but in steady-state, heijunka is preferable. Korytkowski et al. [16] evaluated the performance of an assembly line in a microelectronics factory that required modifications to achieve two objectives: minimization of the average throughput time and of the average work-in-progress. The results showed that appropriate arrangement of heijunka improved either throughput or work-in-progress. In another paper, Korytkowski et al. [17] discussed a multi-product lot-sizing problem for a job shop controlled with a heijunka box using an approach named exponential smoothing. The simulation study of this approach showed not transferring demand fluctuations to the manufacturing system, thus simplifying shop floor management and making production planning more predictable. Renteria-Marquez et al. [18] presented a methodology of modelling accuracy of a production floor, warehouse and material handling system of an automotive assembly facility using a simulation method. The result of the simulation experiment was the identification of a batch size of vehicles with minimum work-in-progress on a production floor and shortest lead time. de la Cruz et al. [19] proposed a Lean model of picking in a warehouse based on heijunka, Kanban, 5S and JIT techniques. The model was subject to assessment using a simulation study. The results demonstrated significant improvements in reducing collector’s load, eliminating wait times for requirements, and increasing the orders served, which led to improvement of the productivity of the picking operation. Rewers [20] used spreadsheet simulation to find the right combination of lot size and production interval in order to obtain the best outcome of order fulfilment and stock levels. Rewers et al. [21] focused on a simulation study on determining variant of the production batch size and production frequency with regard to degree of order processing and machine load.

This article presents the results of research aimed at determining the best variant of the production planning and control system for the production of standard parts included in a customized finished product. Three variants of solutions were adopted:

1. The production of parts is carried out on the basis of production to order, where, after accepting an order for a finished product, the parts manufacturing department receives an order to produce.
2. Production planning of high-speed parts is carried out according to the leveling production, and the rest according to the principle of production to order.
3. The production planning of high-speed parts is carried out according to production leveling, and the remaining parts have a certain appropriate stock level, and their production begins only when apart is taken from the warehouse (pull system).

The article’s primary purpose is concise presentation of the idea of application of heijunka into real production processes. The article is distinct from the literature in two ways: it indicates the need to analyze the structure of a finished product, making it possible to more accurately adapt the production planning and control system to the production of standard parts included in the finished product, and it presents how the documented methodology for implementation of production leveling was adopted, and how simulations were performed using actual production data.

## Materials and methods

**Fig 1:**
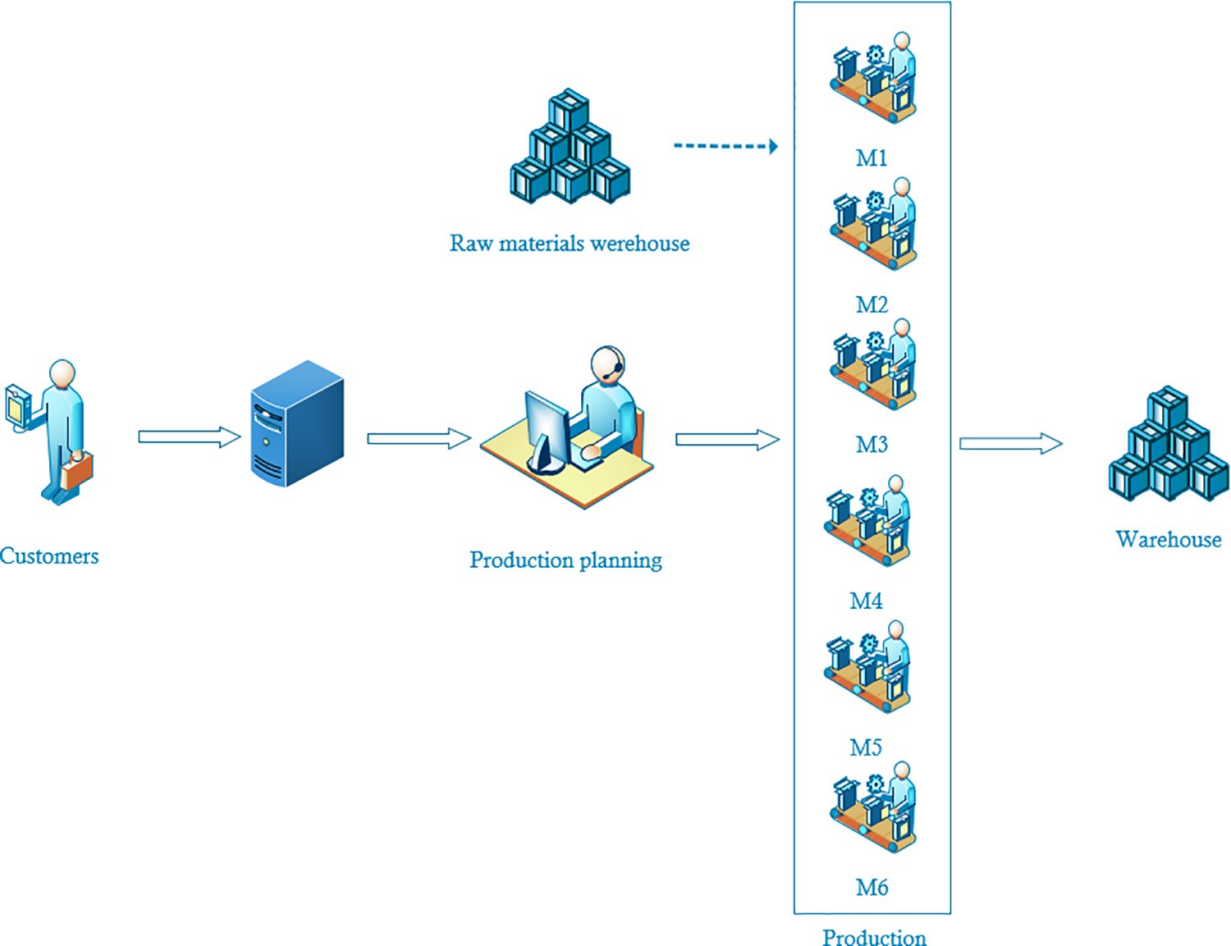
Real production system.

The real system being investigated consists of six machines, a finished products warehouse, a raw materials warehouse and a production planning department (Fig 1).

Customers place an order for finished products via the IT system. The main production planners, on the basis of the bill of materials of the ordered product, outsource the production of parts for the assembly of the finished product to appropriate departments. One of such departments is the department dealing with the production of small-size elements produced in a series. The production of elements takes place on 6 machines, and each product (there are about 400 of them) has an assigned machine on which it can be produced. The planners of the department outsource the production of elements, previously checking the inventory of raw materials and finished products. Currently, product production planning is carried out in large series, adding up various orders for the same parts, which is why there is often a situation where there is no stock of some items in the finished goods warehouse. After receiving the production plan, production workers collect the raw material from the warehouse, and after production is finished, they transfer the finished products to the warehouse, where they are then collected for assembly.

A number of simulation experiments were carried out on the data from the above production system. The obtained data mainly concerned indexes produced, order history of individual elements from a period of one year (number of orders and frequency of orders), production times, changeover times, the technological process of products (including machines on which products were manufactured).

As part of the work, three variants of the planning and control system for the production of standard parts, which make up the finished product assembled according to an assemble-to-order system, were considered. In two variants (variant 2 and 3), the production leveling was applied for products sold in the largest quantities and highest frequency (products with high rotation). These two variants differ in terms of the way of planning and controlling the elements, which are sold rarely and in small quantities (products with low rotation). One variant used production to order (variant 2) while the other variant was controlled by a supermarket (variant 3). Custom production is also intended for variant 1 as a control and planning method for all manufactured products. The individual variants are described below.

**Fig 2:**
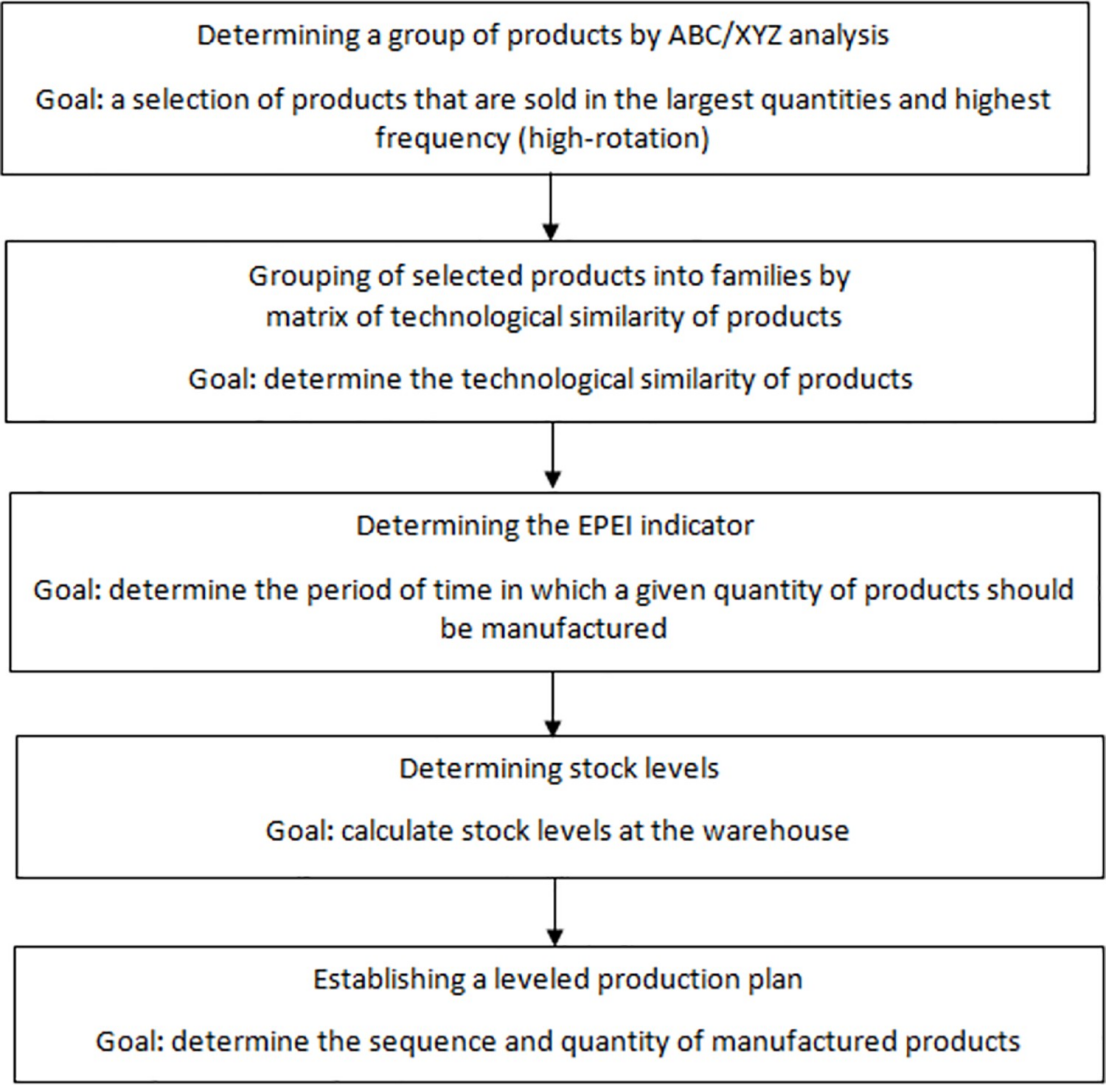
Production leveling algorithm.

Production leveling was carried out in accordance with the algorithm presented in Fig 2.

The simulation results were compared in terms of the execution of production orders (including the execution of orders on time, i.e. when a given part is needed for assembly), the degree of use of the available machine working time and the average waiting time for the manufactured part. Due to the small size of the product and the company’s strategy pointing to the superiority of order timeliness over costs, the data obtained from the system and then when assessing the simulation did not take into account production costs and warehouse costs.

**Fig 3:**
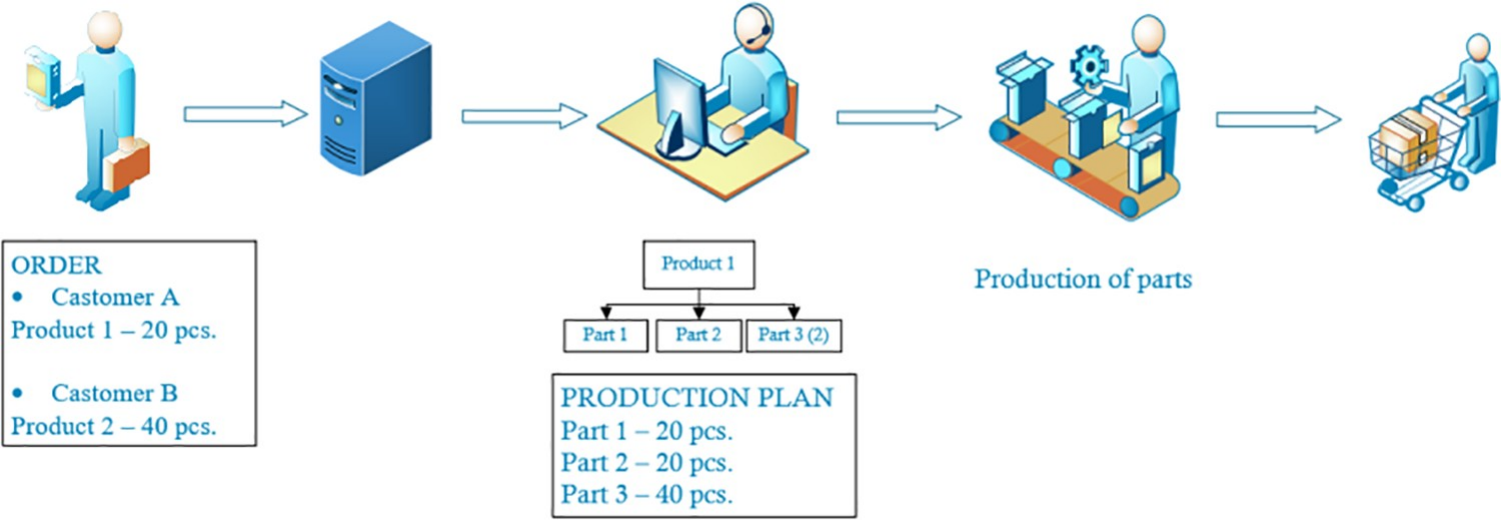
Control method in variant 1.

Variant 1 assumes that production of all parts would be carried out on the basis of production to order, where after receiving an order for the finished product, the demand for parts wouldbe determined based on a bill of materials, i.e. the list of components included in the finished product, which would be synonymous with the production schedule for these parts. Fig 3 shows the control method in variant 1.

**Fig 4:**
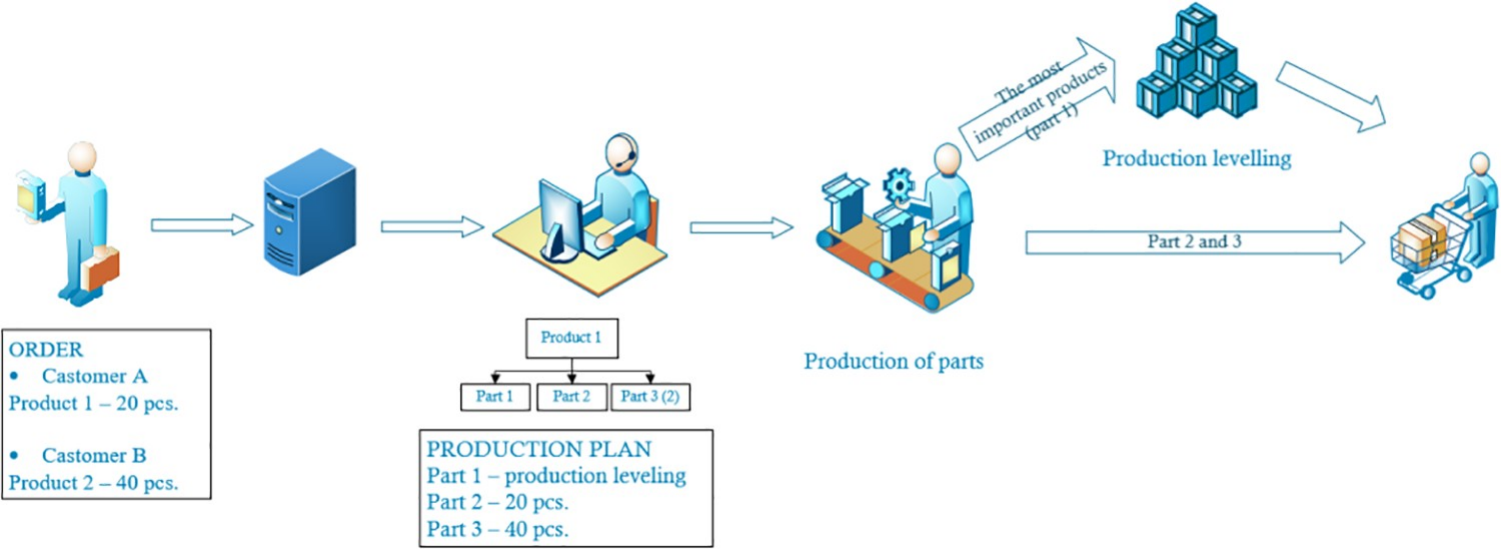
Control method in variant 2.

Variant 2 assumes that for high-rotation parts, i.e. those that are needed for assembly in the largest quantities and most often, production would be carried out in accordance with the production leveling principles, while the remaining parts would be manufactured according to the production to order principles (as in variant 1). This means that for high-rotation parts, a permanent production plan would be established based on one of the production leveling methodologies available in the literature. Parts for further assembly would be taken from the warehouse. Fig 4 shows the control method in variant 2.

**Fig 5:**
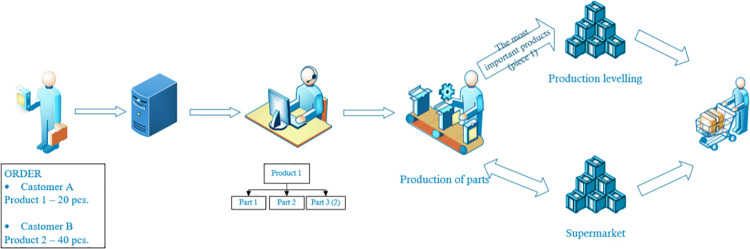
Control method in variant 3.

Variant 3 assumes that for high-rotation products, production would be carried out in accordance with the production leveling rules (as in variant 2), while the remaining parts wouldhave a certain stock level, and their production would start only when the part is left warehouse picked up for assembly. This means that for these parts, the stock level is kept constant in the warehouse, and production would start when the stock level is disturbed. Fig 5 shows the control method in variant 3.

The presented variants were assessed against four criteria:

1. Criterion A—number of completed orders. Total number of customer orders fulfilled during the simulation. The best option is that with the highest number of completed orders.
2. Criterion B—number of orders completed on time. The number of all orders completed within the assumed time, without delays in execution. The best variant is that with the highest number of orders completed on time.
3. Criterion C—average waiting time for an order. The average time needed from the entry of the order to its execution. The best option is that with the lowest average waiting time for the order.
4. Criterion D—the degree of use of the available machine working time. The percentage value of manufacturing, changeover and downtime times. The best option is that with the highest degree of use of the available machine operating time.

In order to identify the variant that best meets all of the indicated criteria, it was decided to use a point method, which awarded the variants with points from 1 to 3, where 3 points were awarded to the variant which met the criterion to the greatest extent, and 1 point awarded to the variant which least fulfilled the criterion. The awarded points were totaled, and the variant with the highest total of points was considered to be the variant that best meets the indicated criteria.

**Fig 6:**
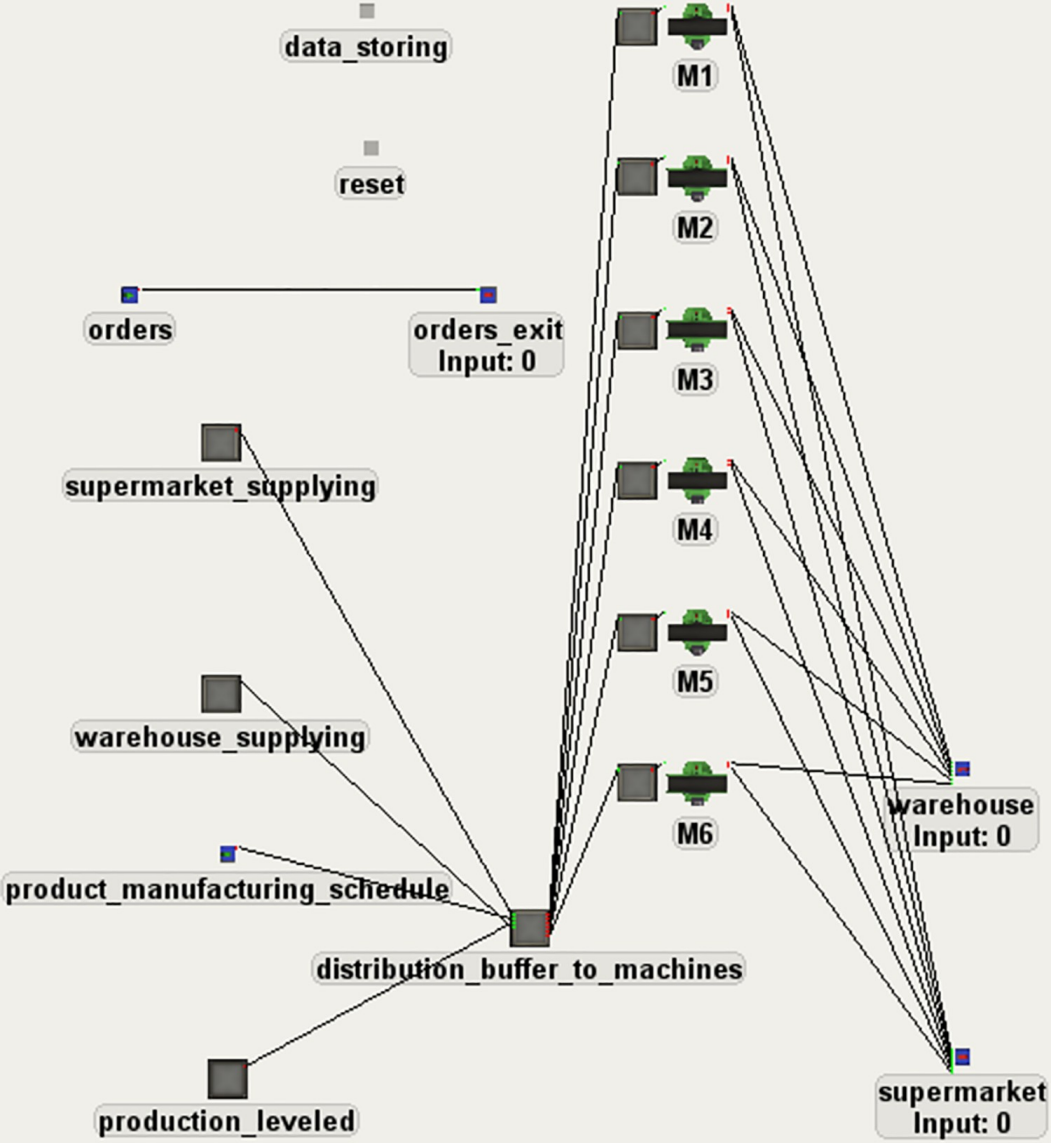
The view of the simulation model.

**Fig 7:**
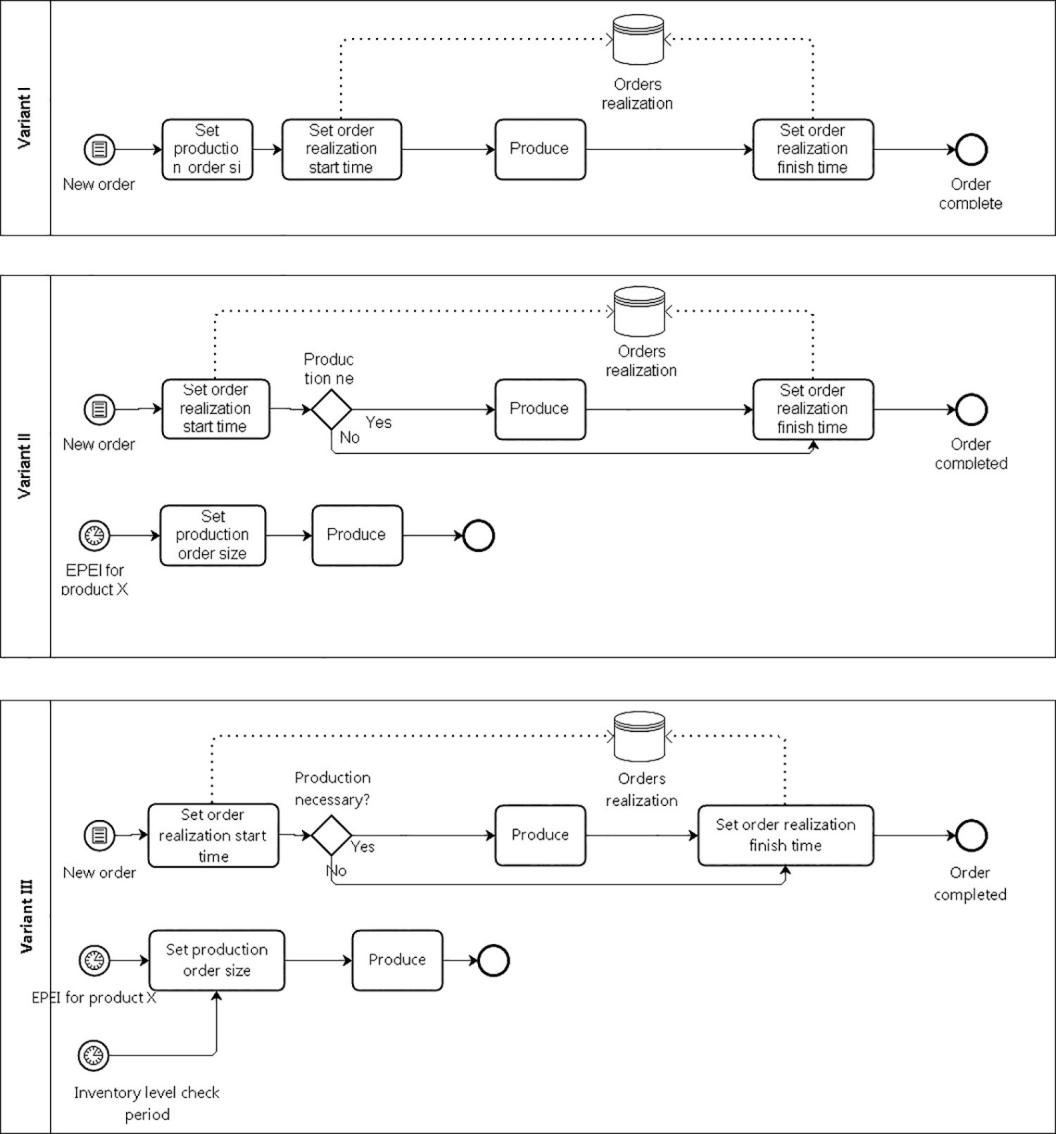
The logic implemented in the analyzed variants.

The analysis of process was conducted using a simulation method. In order to conduct the study, a simulation model was prepared in FlexSim simulation environment (Fig 6). In the model, standard FlexSim objects were used for modelling the input, output and manufacturing processes. The control logic implemented in the model reflected, by appropriate parametrization, each of the three variants being analyzed (Fig 7). Data from a real production system was gathered and used in the model as input data. The input data contained order identification number, order size, order start time and order delivery time. The simulation reflected 2,401 hours of real system time (100 three-shift workdays plus a one-hour time-buffer for process finishing). The simulation model operated in discrete-event simulation. The simulation results were compared in terms of the execution of production orders (including the execution of orders on time, i.e. when a given part is needed for assembly), the degree of use of the available machine working time, and the average waiting time for the manufactured part. The simulation model was initialized by setting the appropriate amount of stock levels, according to the analysis conducted during production leveling (Fig 2). The simulation was assumed to be deterministic, thus one simulation run was performed for each simulated variant.

## Results

The results of the simulation carried out for all three variants, with reference to the four assumed criteria, are presented below.

**Fig 8:**
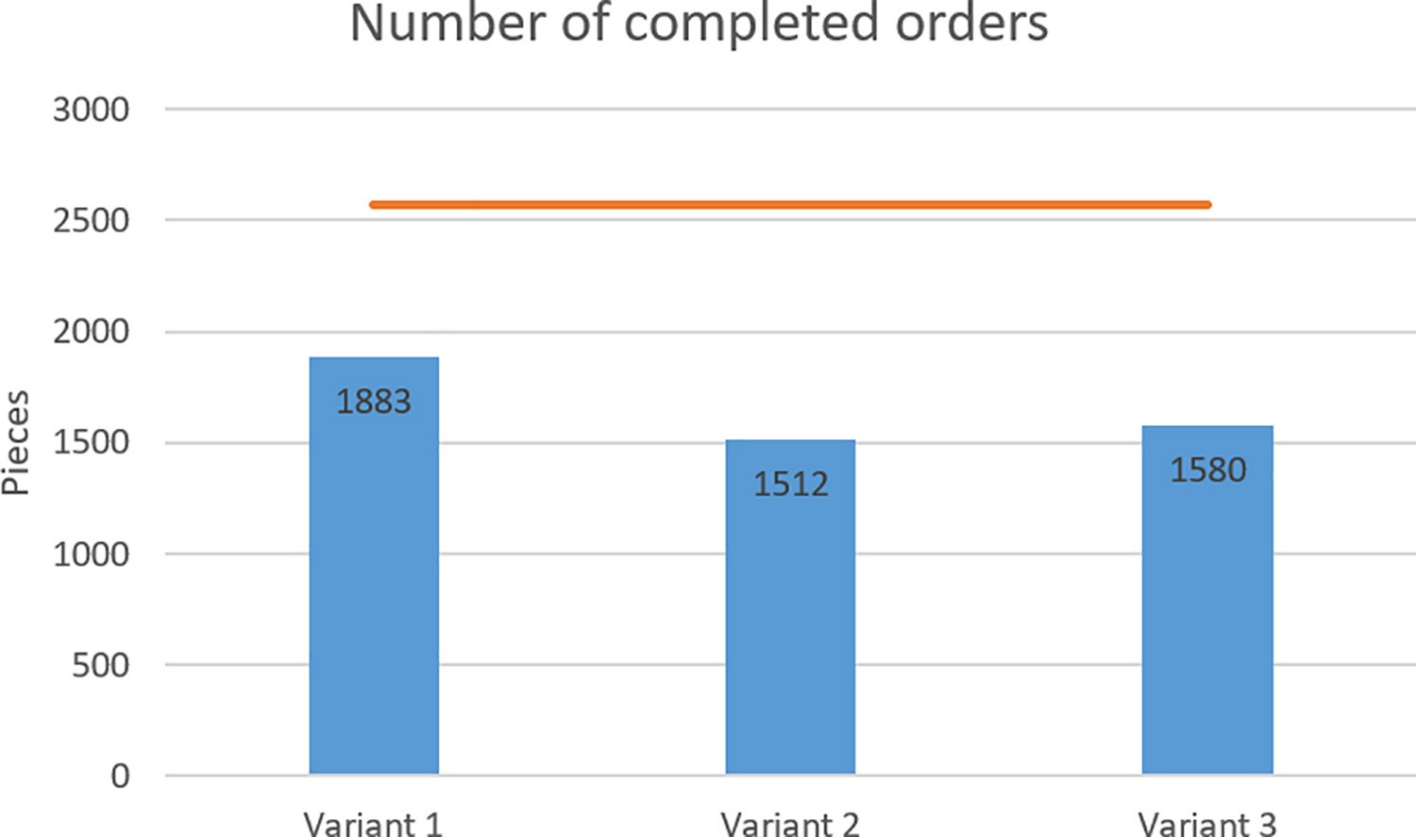
Number of orders completed in individual variants.

In criterion A, the most orders were executed in variant 1 (1,883 orders completed out of 2,570 of all orders), while the least in variant 2 (1,512 orders completed out of 2,570 of all orders). Fig 8 shows the number of completed orders in relation to the number of all orders placed.

**Fig 9:**
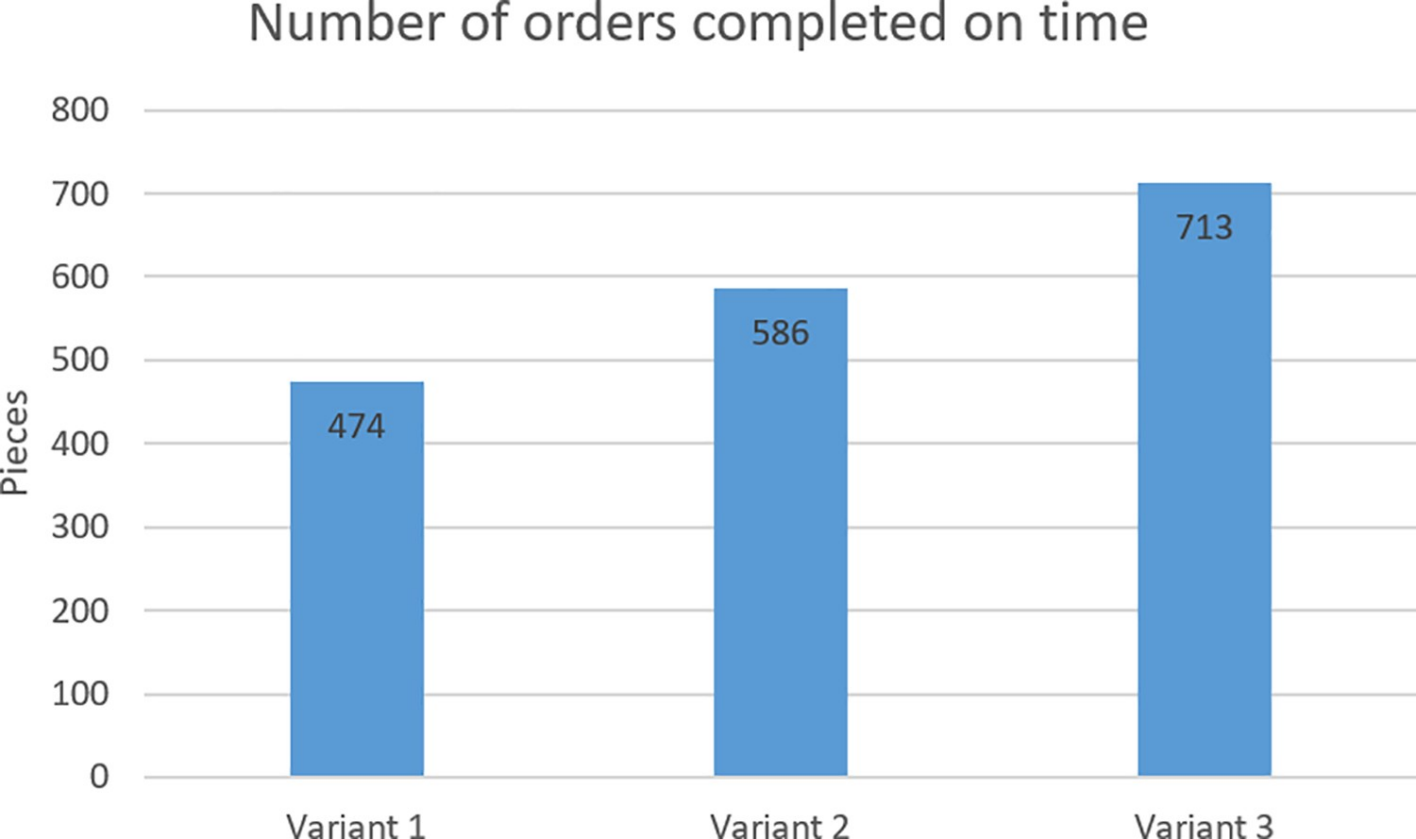
Number of orders completed on time in individual variants.

In criterion B, the most orders were delivered on time in variant 3 (713 orders completed on time), while the least in variant 1 (474 orders completed on time). Fig 9 shows the number of orders completed on time in individual variants.

**Fig 10:**
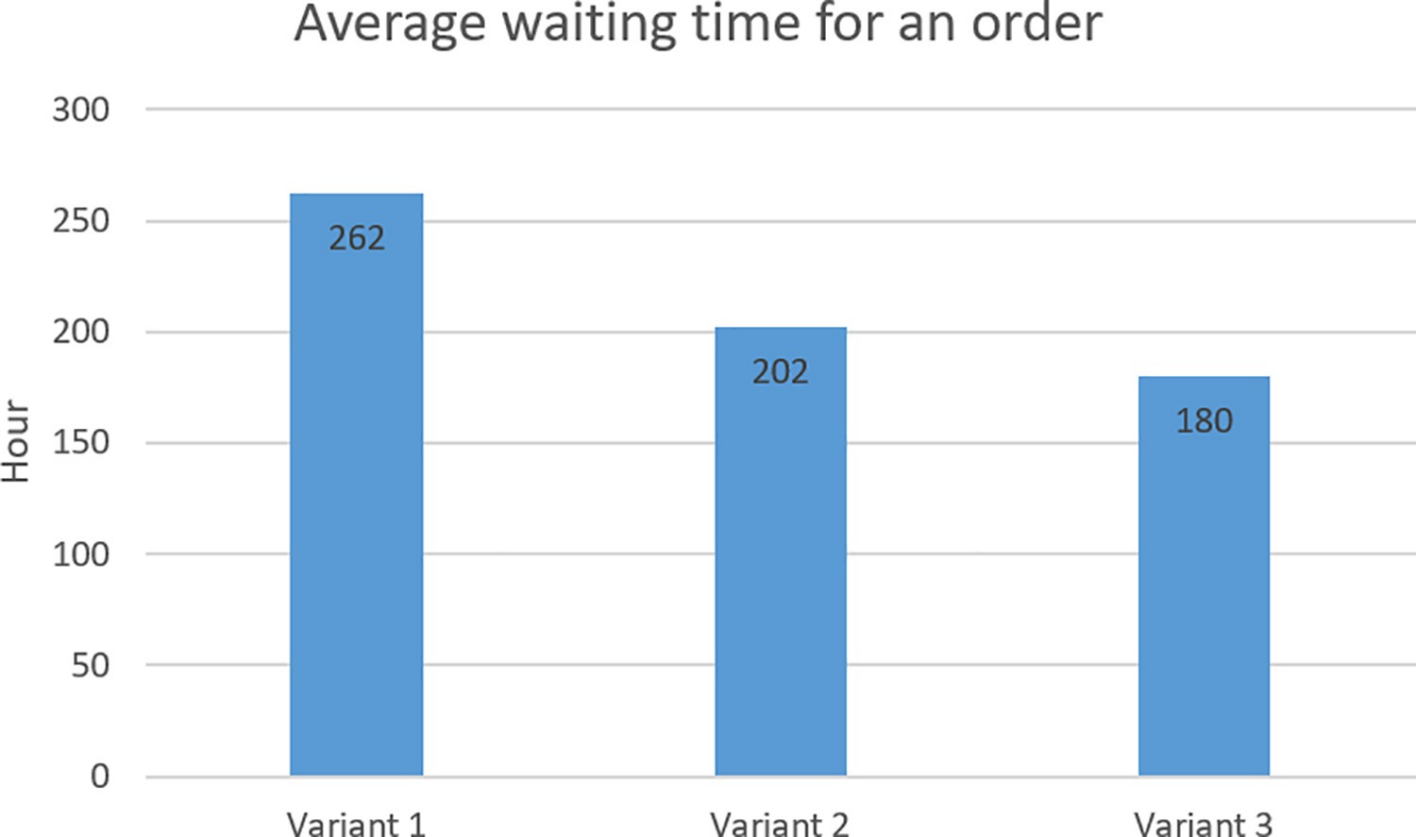
Average waiting time for an order in individual variants.

In criterion C, the fastest orders were delivered in variant 3 (about 180 hours), and the longest in variant 1 (about 262 hours). Fig 10 shows the average waiting time for an order in individual variants.

**Fig 11:**
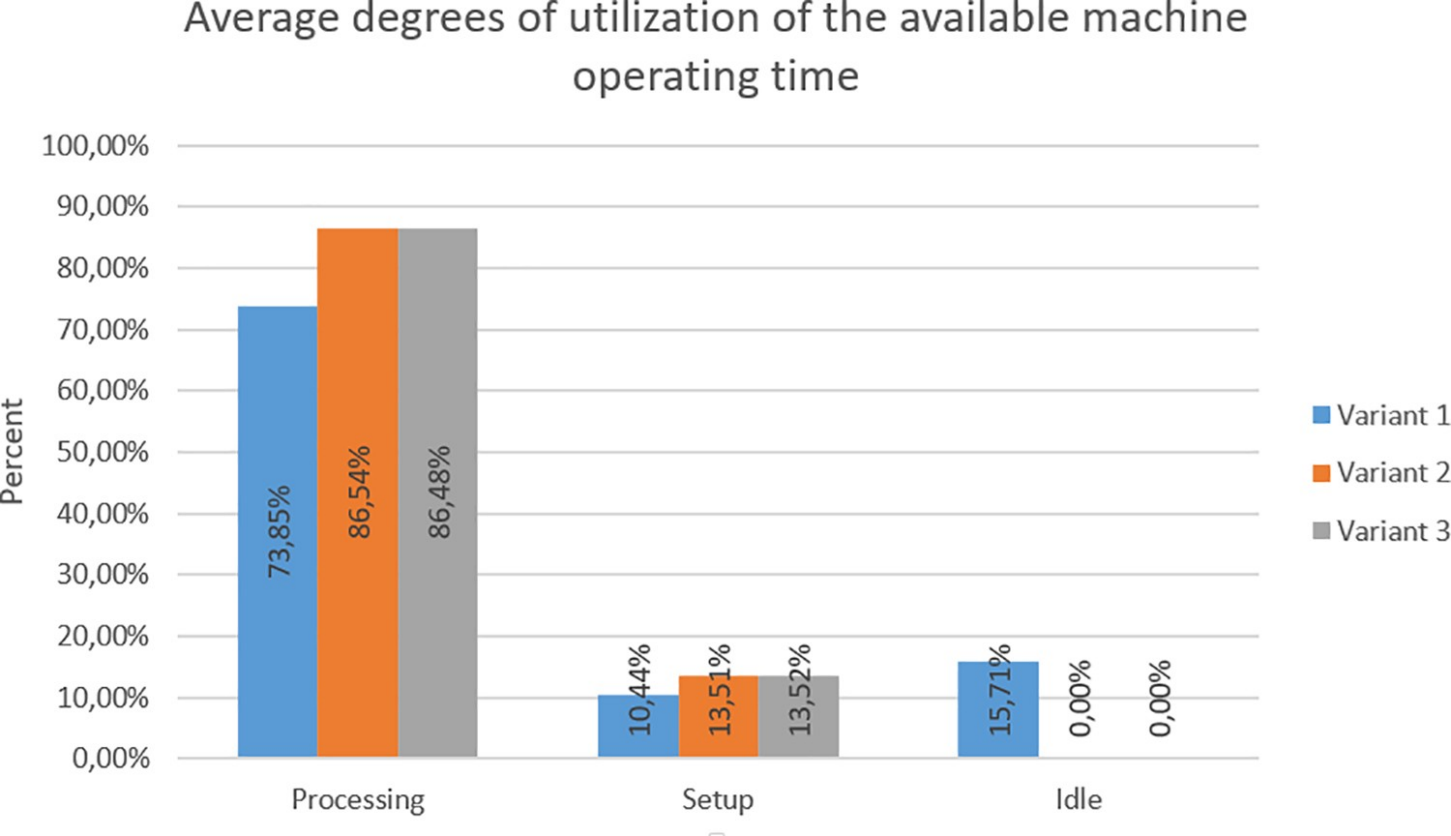
Average degrees of utilization of the available machine operating time in individual variants.

In criterion D, the main assumption was the highest possible machine processing time and the lowest idle time. There was an idle time only in variant 1,thus the processing time is lower. The highest processing time occurred in variant 2, but it was highly similar to the time in variant 3. Taking into account the setup time, it was at a comparable level in all variants. Fig 11 shows the average degrees of utilization of the available machine operating time for each variant.

Table 1 shows the results of the scoring method used to compare the variants. The variant that best met the indicated criteria is variant 3, which assumed the use of production leveling for the most important products, and for the remaining products- production for the supermarket.

**Table 1:** Results of the comparison of variants.

Criterion/Variant	Variant 1		Variant 2		Variant 3	
Criterion A—Number of completed orders	1,883 pieces		1,512 pieces		1,580 pieces	
Points for criterion A	3		2		1	
Criterion B—Number of orders completed on time	474 pieces		586 pieces		713 pieces	
Points for criterion B	1		2		3	
Criterion C—Average waiting time for an order	262 hours		202 hours		180 hours	
Points for criterion C	1		2		3	
Criterion D—Average degrees of utilization of the available machine operating time	Processing	73.85%	Processing	86.54%	Processing	86.48%
	Setup	10.44%	Setup	13.51%	Setup	13.52%
	Idle	15.71%	Idle	0.00%	Idle	0.00%
Points for criterion D	1		3		2	
Sum of points	6		8		10	

## Conclusions

This article presents simulation studies concerning the determination of the variant of the production planning and control system that meets the assumed criteria to the greatest extent. The presented example concerns the production of standard parts included in the finished product manufactured according to an assemble-to-order system. As shown by the research, the best variant, assessed according to the adopted scoring method, was variant 3, which assumed the use of production leveling for products sold in the largest quantities and highest frequency, and for the remaining products- production for the supermarket. This variant received the highest score in two criteria—criterion B and criterion C, and the average score in the other two criteria.

Use of a simulation method for the presented problem allows the matching of the way of process organization and control, and takes into account the character of a particular production process. It also allows to calibrate the key parameters for obtaining the most profitable results for the process owner.

In further research, the authors will try to determine which process parameters (product completion time, changeover time, machine efficiency, product procurement method, etc.) of the known production planning and control methods will produce the best results in terms of time-based orders and the degree of use of machines. In addition, by conducting a series of simulations and tests, the authors will try to generalize in what conditions, and under what parameters of the production process, production leveling gives the best results in terms of order fulfillment and degree of machine use. According to the literature analysis, the effectiveness of production leveling can be checked with many measures. As reported in the literature on the subject, production leveling affects, among others, operational efficiency, shortening the time of executing orders, or reducing warehousing costs. In its assumptions, production leveling should also lead to an increase in the quality of customer service or an increase in the competitiveness of production companies. On the other hand, through the production of a constant and unchanging sequence of products for a certain period of time, it leads to a reduction in the flexibility of production, which in the current economic situation is considered to be one of the most important factors determining the company’s situation on the market. Other measures and determinants of production leveling can also be found in other works. However, no detailed information or research on the impact of production leveling on the degree of order fulfillment and the degree of machine utilization was found.

## Supporting information

S1 Data Simulation_parameters-orders.xlsx–simulation model parameters–orders. (XLSX)

S2 Data Simulation_parameters-products.xlsx–simulation model parameters–products. (XLSX)

## References

[1] Liker JK, Meier D. The Toyota way field book: A practical guide for implementing Toyota’s 4Ps. 1st ed. London: McGraw-Hill; 2005.

[2] Wang Y, Ma HS, Yang JH, Wang KS. Industry 4.0: A way from mass customization to mass personalization production. Advances in Manufacturing. 2017 Nov 28; 5:311–20. doi: 10.1007/s40436-017-0204-7

[3] Zawadzki P, Żywicki K. Smart product design and production control for effective mass customization in the Industry 4.0 concept. Management and Production Engineering Review. 2016; 7(3): 105–12. doi: 10.1515/mper-2016-0030

[4] Mukherjee K, Sarkar B, Bhattacharjya A. Supplier’sselection strategy for mass customization. International Conference on Computers&Industrial Engineering. 2009; 892–5. doi: 10.1109/ICCIE.2009.5223861

[5] Bednar S, Modrak V, Mass customization and its impact on assembly process’ complexity. International Journal for Quality Research. 2014 Sep 01; 8(3): 417–30.

[6] Dennis P. Lean Production Simplified. New York: Productivity Press; 2007.

[7] Liker JK. The Toyota Way. New York: McGraw-Hill;2004.

[8] Rewers P, Hamrol A, Żywicki K, Kulus W, Bożek M. Production leveling as an effective method for production flow control—experience of polish enterprises. Procedia Engineering. 2017 Apr 18; 182: 619–26. doi: 10.1016/j.proeng.2017.03.167

[9] Rewers P, Trojanowska J, Diakun J, Rocha A, Reis LP. A study of priority rules for a levelled production plan. In: Hamrol A, Ciszak O, Legutko S, Jurczyk M, editors. Advances in Manufacturing. Lecture Notes in Mechanical Engineering. Cham: Springer; 2018. pp. 111–120.

[10] Żywicki K, Rewers P, Bożek M. Data analysis in production levelling methodology. In: Rocha Á, Correia A, Adeli H, Reis L, Costanzo S, editors. Advances in Intelligent Systems and Computing. Cham: Springer; 2017. pp.460–8.

[11] Mourtzis D, Doukas M, Bernidaki D. Simulation in manufacturing: Review and challenges. ProcediaCIRP. 2014 Dec 10; 25: 213–29. doi: 10.1016/j.procir.2014.10.032

[12] Pedgen CD, Shannonm RE, Sadowski RP. Introduction to simulation using SIMAN. NewYork: McGraw Hill; 1995.

[13] Matzka J, Di Mascolo M, Furmans K. Buffer sizing of a Heijunka Kanban system. Journal of Intelligent Manufacturing. 2012 Feb; 23:49–60. doi: 10.1007/s10845-009-0317-3

[14] Lage Junior M, Filho M, Variations of the kanban system: Literature review and classification. International Journal of Production Economics. 2010; 125(1):13–21. doi: 10.1016/j.ijpe.2010.01.009

[15] Runkler TA. Controlling discrete manufacturing processes using Kanban and Heijunka approaches. 9th IEEE International Conference on Industrial Informatics. 2011. pp. 181–86. doi: 10.1109/INDIN.2011.6034859

[16] Korytkowski P, Wisniewski T, Rymaszewski T. Multivariate simulation analysis of production leveling (heijunka)—a case study. 7th IFAC Conference on Manufacturing Modelling, Management, and Control International Federation of Automatic Control. Saint Petersburg, Russia. June 19–21, 2013.

[17] Korytkowski P, Grimaud F, Dolgui A. Exponential smoothing for multi-product lot-sizing with heijunka and varying demand. Management and Production Engineering Review. 2014; 5(2): 20–6. doi: 10.2478/mper-2014-0013

[18] Renteria-Marquez IA, Almeraz CN, Tseng TL, Renteria A. A Heijunka study for automotive assembly using discrete-Event Simulation: a Case Study. Proceedings of the 2020 Winter Simulation Conference. 2020. pp. 1641–1651. doi: 10.1109/WSC48552.2020.9383927

[19] de la Cruz H, Altamirano E, del Carpio C. Lean model to reduce picking time delays through Heijunka, Kanban, 5S and JIT in the construction sector. 18th LACCEI International Multi-Conference for Engineering, Education, and Technology. 27–31 July 2020. Virtual Edition., doi: 10.18687/LACCEI2020.1.1.92

[20] Rewers P, Planning the inflow of products for production levelling. International Scientific Journal "Machines. Technologies. Materials.". 2019; 13(10): 439–42.

[21] Rewers P, Czaja M, Janczura K, Diakun J. Determination of the production frequency and batch size for the manufacturing process. In: Tonkonogyi V. et al. editors. Advanced Manufacturing Processes II. InterPartner 2020. Lecture Notes in Mechanical Engineering. Cham: Springer. 2021.

